# The relation between mental health, homosexual stigma, childhood abuse, community engagement, and unprotected anal intercourse among MSM in China

**DOI:** 10.1038/s41598-018-22403-9

**Published:** 2018-03-05

**Authors:** Yaxin Zhu, Jie Liu, Yucun Chen, Ruochen Zhang, Bo Qu

**Affiliations:** 1 Department of Social Medicine, School of Public Health, China Medical University, Shenyang, Liaoning Province P.R. China; 2 Department of Health Statistics, School of Public Health, China Medical University, Shenyang, Liaoning Province P.R. China; 3 Dalian Center for Disease Control and Prevention, Dalian, Liaoning Province P.R. China

## Abstract

The aim of the study was to explore the relation of various factors with unprotected anal intercourse (UAI) and provide some insight for HIV intervention on Chinese men who have sex with men (MSM). The current cross-sectional study recruited 365 MSM in Dalian, China. More than half of the respondents (117 respondents, 51.8% of the sample) had engaged in UAI. The multivariable logistic regression model suggested that poorer mental health (AOR: 7.16; 95% CI: 3.14–16.31), self-stigma (AOR: 1.53; 95% CI: 1.00–2.34), and experience(s) of physical abuse in childhood (AOR: 5.85; 95% CI: 1.77–19.30) were significantly and positively related to UAI. Community engagement was negatively associated with UAI (p < 0.05). It appears it is necessary to incorporate mental health services, eliminate the stigma against homosexuality, and facilitate MSM-related community engagement into intervention strategies to prevent UAI among Chinese MSM. Targeted UAI interventions in the subgroup with a history of childhood physical abuse should also be of great concern.

## Introduction

Due to HIV prevention efforts, the overall prevalence of HIV in China remains low; however, the rate of HIV infection has increased dramatically in the men who have sex with men (MSM) population^1^. It was estimated that sexually-active MSM accounted for 2–4% of the total sexually-active men in China^2^. However, in 2016, MSM accounted for 27.6% (34,399 cases) of the total estimated new diagnoses of HIV/AIDS in China^3^. In addition, the percentage was more than 10 times higher than that in 2006 which was 2.5%^1^. In 2014, the percentage of people living with HIV in the MSM population was the highest among all the high-risk AIDS groups in China (7.7%)^1^. A leading risk behaviour of HIV infection among MSM is unprotected anal intercourse (UAI), in which approximately half of Chinese MSM have reported participating in^1,4,5^. MSM might also have unprotected sex with women which could also contribute to the rate of HIV infection within the general population^1^. As a key at-risk population for HIV infection, MSM require focused attention for HIV prevention^1,4^. In addition, intervention efforts aimed at reducing UAI may facilitate a reduction in HIV transmission^6^. The development of effective interventions should also take into account multiple related factors that contribute to HIV high-risk behaviour within the MSM population^5,6^.

Taken as a whole, individual, psychosocial, social, and structural factors mutually amplify the risk of HIV infection among MSM^7,8^. The reciprocal relations among stigma, mental health, and HIV-related risk behaviour have been reported among MSM in the United States^9,10^. Homosexuality-related stigma is prevalent in China owing to the fact that homosexuality is against traditional Chinese values, which emphasizes family continuity^11^. Although the positive relation between homosexual stigma and UAI has been well documented in previous studies^10,12–14^, few studies have reported this in China^11^. Mental distress is also a serious problem among MSM and is more prevalent among MSM than among heterosexual men^15,16^. Compared with a general population of men, MSM in the U.S. Urban Men’s Health Study reported a 2.7-fold greater risk of distress and depression^16^. A growing body of research has demonstrated that poor mental health is a contributing factor to HIV risk behaviour among MSM^17,18^.

Community engagement refers to the process of working collaboratively with groups of people connected by issues influencing their lives^19^. Among HIV-infected populations, community engagement is positively associated with psychosocial and physical health^20^. As a socially marginalized population, MSM may also benefit from community engagement^21^. Community-based organizations (CBOs), which are significant vehicles for community engagement for MSM, play a more cost-effective role in the AIDS response than government facilities among Chinese MSM as they offer increased access to available HIV services^22,23^. However, there is little information available in the literature about the association between community engagement and HIV risk behaviour in Chinese MSM.

Several recent studies have suggested that childhood abuse is a risk factor for HIV sexual risk behaviour among MSM^24,25^. In addition, a substantial proportion of MSM have experienced childhood abuse^26^. Childhood abuse refers to physical, sexual, or psychological mistreatment, especially by a parent or other caregiver^25^. Studies have focused on the association of childhood sexual abuse (CSA) and HIV risk behaviour among MSM; those who have experienced CSA were more likely to engage in sexual risk behaviour^27,28^. Besides CSA, little is known about the influence of other types of childhood abuse on HIV risk behaviour among the MSM population^24^. Additionally, it has been reported that physical abuse is much more common than sexual abuse during childhood^24,29^.

In this paper, we explore the association of various factors with UAI, including homosexuality-related stigma, mental health, history of childhood abuse (both physical abuse and mental abuse), community engagement, and HIV knowledge. This insight into UAI and various associated factors may help facilitate the development of targeted HIV intervention measures among Chinese MSM.

## Results

### Participant characteristics

Of the 365 individuals who met the inclusion criteria and agreed to provide written informed consent, 342 (93.7%) completed the survey. The mean age of the 342 participants was 28.48 years, with a standard deviation of 7.18 years. Within the study sample, 29.2% of the participants only completed middle school or below while 42.7% completed secondary technical school or high school. Additionally, 77.2% of the participants had a monthly income of less than 3000 yuan. Most of the surveyed MSM were single (262, 76.6%).

For the anal sex role, 142 (41.5%) reported themselves as undertaking the insertive anal sex role with their male partners, 106 (31.0%) reported themselves as the receptive anal sex role, and the rest (94, 27.5%) were versatile. More than half of the respondents (117, 51.8%) had engaged in UAI. Only two of the basic characteristics (age and education level) were significantly associated with UAI. The associations of socio-demographic and anal sex role characteristics with UAI are detailed in Table 1.

**Table 1:** Simple logistic analysis of socio-demographic characteristics and anal sex role associated with UAI among Chinese MSM (n = 342).

Characteristic	Total n (%)	OR (95% CI)
**Age (years)**		
<=25	140 (40.9%)	1
26–35	147 (43.0%)	0.56 (0.35–0.90)*
36–44	39 (11.4%)	0.61 (0.30–1.24)
>=45	16 (4.7%)	2.12 (0.65–6.91)
**Education level**		
Middle school and below	100 (29.2%)	1
Secondary technical school or high school	146 (42.7%)	0.43 (0.25–0.72)*
College and above	96 (28.1%)	0.61 (0.35–1.08)
**Monthly income (yuan)**		
<2000	109 (31.9%)	1
2000–3000	155 (45.3%)	1.17 (0.72–1.91)
>3000	78 (22.8%)	1.50 (0.83–2.69)
**Marital status**		
Single	262 (76.6%)	1
Married	51 (14.9%)	1.24 (0.68–2.26)
Divorced	29 (8.5%)	1.93 (0.86–4.31)
**Anal Sex role**		
Insertive	142 (41.5%)	1
Receptive	106 (31.0%)	0.93 (0.56–1.53)
Versatile	94 (27.5%)	1.41 (0.83–2.39)

^*^p < 0.05; OR: odds ratio; CI: confidence interval; MSM: men who have sex with men.

### Mental health

In our study sample, 169 respondents (49.4%) had psychological problems as per the cut-off point of four or greater on the GHQ-12. The distribution of the GHQ-12 item scores is presented in Table 2. The most prevalent mental health problems reported by the participating MSM were seldom or never feeling reasonably happy (170, 49.7%), usually or more than usually under stress (169, 49.4%), and thinking of oneself as worthless (154, 45.0%).

**Table 2:** Descriptive statistics of GHQ-12 among Chinese MSM (n = 342).

	Seldom/Not at all	Usual/More than usual
Able to concentrate*	54 (15.8%)	288 (84.2%)
Lost much sleep	275 (80.4%)	67 (19.6%)
Playing a useful part*	115 (33.6%)	227 (66.4%)
Capable of making decisions*	133 (38.9%)	209 (61.1%)
Under stress	173 (50.6%)	169 (49.4%)
Could not overcome difficulties	250 (73.1%)	92 (26.9%)
Enjoy normal activities*	55 (16.1%)	287 (83.9)
Face up to problems*	52 (15.2%)	290 (84.8)
Feeling unhappy and depressed	189 (55.3%)	153 (44.7%)
Losing confidence	191 (55.8%)	151 (44.2%)
Thinking of oneself as worthless	188 (55.0%)	154 (45.0%)
Feeling reasonably happy*	170 (49.7%)	172 (50.3%)

^*^Six positively worded items; MSM: men who have sex with men.

### Homosexual stigma

The mean of SSS-S was 2.26 ± 0.91. Respondents obtained higher scores on the affective dimension (2.46 ± 0.80) than on the behavioural (2.14 ± 1.03) and cognitive (2.16 ± 1.01) dimension. In addition, the item “fearing that others would know that they are MSM” scored the highest (3.01 ± 1.09). The details of SSS-S are displayed in Table 3.

**Table 3:** Descriptive statistics of SSS-S among Chinese MSM (n = 342).

**Domain and item**	**Mean ± SD**
Affective dimension	2.46 ± 0.80
I fear that others would know that I am a MSM.	3.01 ± 1.09
I feel helpless because I am a MSM.	2.21 ± 1.03
I feel uncomfortable because I am a MSM.	2.18 ± 1.02
Behavioural dimension	2.14 ± 1.03
I dare not to make new friends lest they find out that I am a MSM.	2.09 ± 1.06
I estrange myself from others because I am a MSM.	2.16 ± 1.05
I avoid interacting with others because I am a MSM.	2.18 ± 1.05
Cognitive dimension	2.16 ± 1.01
The identity of being a man who has sex with men taints my life.	2.14 ± 1.03
My identity as a MSM incurs inconvenience in my daily life.	2.17 ± 1.03
My identity as a MSM is a burden to me.	2.17 ± 1.04
Mean Self-stigma	2.26 ± 0.91

MSM: men who have sex with men; SD: standard deviation.

### Factors associated with UAI

Table 4 shows the results of simple and multivariable logistic regression analysis of associated factors with UAI. The simple logistic regression results showed that all the explored variables were significantly associated with UAI among MSM (p < 0.05). After adjusting for the significant socio-demographic variables (age and education level) in the multivariable logistic regression model, the three variables that were significantly and positively associated with UAI were poorer mental health (AOR: 7.16; 95% CI: 3.14–16.31), self-stigma (AOR: 1.53; 95% CI: 1.00–2.34) and the experience of physical abuse in the childhood (AOR: 5.85; 95% CI: 1.77–19.30). The two significantly and negatively associated factors were the number of CBO affiliations (number = 1: AOR = 0.08; 95% CI: 0.04–0.17; number> = 2: AOR = 0.04; 95% CI: 0.01–0.09; reference group: number = 0) and the frequency of community activity engagement (occasionally: AOR = 0.48; 95% CI: 0.21–1.06; always: AOR = 0.19; 95% CI: 0.08–0.45; reference group: never) (Table 4).

**Table 4:** Simple and multivariable logistic regression analysis of factors associated with UAI among Chinese MSM (n = 342).

**Factor**	**Total n (%)**	**COR (95% CI)** ^**a**^	**AOR (95% CI)** ^**b**^
**Mental health**			
GHQ <4	173 (50.6%)	1	1
GHQ >=4	169 (49.4%)	2.09 (1.36–3.21)*	7.16 (3.14–16.31)*
**SSS-S**			
Affective dimension^c^	—	1.58 (1.19–2.08)*	—
Behavioural dimension^d^	—	1.44 (1.16–1.78)*	—
Cognitive dimension^e^	—	1.45 (1.17–1.80)*	—
Mean Self-stigma	—	1.53 (1.20–1.96)*	1.53 (1.00–2.34)*
**Childhood abuse**			
Psychological abuse			
No	299 (87.4%)	1	1
Yes	43 (12.6%)	2.11 (1.07–4.16)*	0.71 (0.26–1.95)
Physical abuse			
No	306 (89.5%)	1	1
Yes	36 (10.5%)	3.69 (1.63–8.35)*	5.85(1.77–19.30)*
**HIV knowledge**	—	0.73 (0.57–0.93)*	0.74 (0.51–1.08)
**Community engagement**			
Number of CBO affiliations			
0	95 (27.8%)	1	1
1	169 (49.4%)	0.16 (0.09–0.30)*	0.08 (0.04–0.17)*
>=2	78 (22.8%)	0.06 (0.03–0.13)*	0.04 (0.01–0.09)*
Frequency of community activity engagement			
Never	89 (26.0%)	1	1
Occasionally	122 (35.7%)	0.58 (0.33–1.02)	0.48 (0.21–1.06)
Always	131 (38.3%)	0.35 (0.20–0.61)*	0.19 (0.08–0.45)*

^a^Simple logistic regression model. ^b^Multivariable logistic regression model. SSS-S: Self-Stigma Scale–Short Form; MSM: men who have sex with men; CBO: community-based organization; COR: crude odds ratio; CI: confidence interval; AOR = adjusted odds ratio, odds ratio adjusted by significant background variables (educational level and marital status).

*p < 0.05.

## Discussion

Among all the related factors in our study, UAI was best associated with poorer mental health (AOR: 7.16; 95% CI: 3.14–16.31) among MSM. Other studies conducted in the United States and India have also found that mental factors, such as depression and substance use, are associated with sexual risk taking among MSM^30–32^. In addition, the combination of prevention efforts of behavioural interventions and mental health treatment has been demonstrated to improve the effectiveness of reducing HIV transmission risk behaviour^33,34^. Furthermore, approximately half of the participants (49.4%) in our study had mental health problems. Storholm ED *et al*. implied that MSM may be more likely to seek mental health services than their heterosexual counterparts because they suffer higher rates of mental health problems^35^. However, the EXPLORE study in the US found that only 9.71–23.85% of MSM in different age groups are receiving psychological counselling^36^. The low prevalence of mental health service utilization may be explained by the fact that MSM are afraid of discussing their sexual orientation openly or the lack of MSM-friendly medical services^35^.

Internalized homosexual stigma is a big psychosocial problem for Chinese MSM^11^. In addition, our results showed that the self-stigma against their sexual minority status was significantly related to UAI (p < 0.05) among MSM, which was consistent with the findings of studies conducted in India, Uganda, and Vietnam^12–14^. Choi KH *et al*. argued that usage of social support coping and avoidant coping strategies could help to mediate and lessen the direct relation between MSM stigma and HIV risk^37^. Mental health and psychosocial problems frequently co-occur among MSM, and prevention strategies addressing co-occurring risk factors that interact to produce a heightened HIV risk could improve the efficacy of current HIV behaviour interventions^12,33^.

We found that 10.5% of MSM in this study had experienced childhood physical abuse. In addition, MSM in our study who had a history of childhood physical abuse were more likely to engage in UAI (AOR: 5.85; 95% CI: 1.77–19.30). A population-based study in Washington state showed that for men, childhood physical abuse was related to a 3-fold increase in HIV high-risk behaviours (OR: 3.2; 95% CI: 1.3–7.9), including unprotected anal sex^38^. Schilder AJ *et al*. suggested that the potential pathways linking childhood physical abuse to UAI might be that childhood physical abuse placed MSM at an increased risk of UAI by affecting their motivation, cognitive and behavioural coping strategies, risk appraisal, and ultimately, HIV risk behaviour^25^. As noted in other studies, other HIV risk behaviours are also associated with higher rates of childhood physical abuse among MSM, such as having multiple sex partners and using methamphetamine^24,26^. In addition, a previous study suggested that it may be helpful to conduct family-based interventions on childhood abuse focusing on psychoeducation, enhancing communication between family members, and coping skills^39^. Thus, efforts to enhance the effectiveness of interventions for UAI among MSM should consider the possibility of a history of childhood physical abuse^25,26^.

The results also showed that community engagement was negatively related to UAI among MSM (p < 0.05). The involvement of MSM-related CBOs has shown to facilitate AIDS interventions, such as increased HIV testing rates, retention in care, and adherence to treatment^19,22^. However, Halkitis PN *et al*. implied that affiliation to the MSM community may either protect against or increase the HIV risk^8^. Access to a MSM community in the absence of norms promoting safer sexual behaviours may lead to greater sexual risk taking^8,40^. CBOs have a limited role in China’s AIDS response due to insufficient funds, an unfavourable policy environment, and an incomplete working mechanism^1,19^. In addition, in 2014, the number of CBOs involved in the AIDS response decreased because certain international cooperative AIDS projects were over. Therefore, the Chinese government should provide the funding necessary to create a quality assurance system to support their involvement in the AIDS response^1^. In Sichuan Province, China, an official evaluation system was established to assess the capabilities of CBOs in the AIDS response, with the aim of improving standard management^1^.

There are limitations to this study that should be acknowledged. First, all participants were enrolled by convenience in one city of China. This may limit the generalisability of the findings. Additionally, most of the participants were of low socioeconomic status (SES). As such, this may result in a difference of associations (direction and magnitude) between variables with other studies of more representative samples. Further research should involve more people of middle to high SES. Second, 6.3% of the initially recruited MSM refused to finish the survey, which may lead to an underestimate of the study outcomes as MSM with greater internalized stigma may be less likely to participate. Third, the anal sex role of UAI was also an important predictor, and its association with the study measures should be explored in future research. Fourth, we just used 2 self-designed questions to assess community engagement, and the reliability of this measure was moderate. Some standardized instruments should be used to make the results more reliable. Last, its cross-sectional design prevents us from establishing a causal relation between UAI and the risk factors examined.

Our findings show that multiple factors are related to the occurrence of UAI among MSM who are at risk for HIV infection. These findings have important implications for AIDS prevention and intervention programs. It appears necessary to incorporate mental health services, eliminate homosexual stigma, and facilitate MSM-related community engagement into future intervention strategies to prevent UAI among Chinese MSM. Special attention should also be given to the subgroup with a history of childhood physical abuse, as they appear to be more likely to engage in UAI. These findings support the need for an integrated UAI intervention for MSM.

## Methods

### Ethics statement

The Bioethics Advisory Commission of China Medical University approved this study. The participants were informed about the purpose of the study and, prior to the start of the study, were assured that their privacy would be protected. Informed consent was obtained from all individual participants included in the study. All methods in this study were performed in accordance with the relevant guidelines and regulations.

### Data availability statement

All data generated or analysed during this study are included in this published article.

### Study population and procedures

A convenience sample was recruited from Dalian, in Liaoning province, which is situated in the northeast region of China. The inclusion criteria were that participants had anal intercourse with men in the previous 6 months, were 18 years of age or older, were assigned as male at birth and were without cognitive impairment. In total, 365 participants were recruited from gay bars, gay saunas, and parks between January and March 2016 with the support of local Centres for Disease Control and Prevention (CDC) branches. All participants completed a self-administered questionnaire voluntarily.

### Measures

#### Socio-demographic information

Participants reported their age, highest level of education, monthly income, and current relationship status.

#### Unprotected anal intercourse

One question assessed for the experience of UAI (yes *vs*. no). UAI was defined as having had at least one episode of anal intercourse with any male sex partner without a condom in the past 6 months.

#### Anal Sex role

Participants were asked to report their anal sex role. It was measured with one question “what’s your usual anal sex role with male partners in your life?” Response categories were “exclusively receptive”, “mostly receptive, sometimes insertive”, “versatile”, “mostly insertive, sometimes receptive”, and “exclusively insertive”. Those responding “exclusively receptive” or “mostly receptive sometimes insertive” were coded as receptive. Those responding “exclusively insertive” or “mostly insertive sometimes receptive” were coded as insertive and the rest as “versatile”.

#### Mental health

A short version of the General Health Questionnaire (GHQ-12)^41^ was used in this study. It is a psychometric screening tool used to identify mental health problems experienced by an individual in the past few weeks. It contains 12 items rated on a 4 point scale, and we used a bi-modal scoring method. The responses of “less than usual” and “no more than usual” were scored equally at 0 point, and “rather more than usual” and “much more than usual” were both scored as 1 point^41^. All item scores were totalled to determine the final GHQ-12 score. A score of 4 or greater was considered indicative of a psychological problem^41^. The Cronbach’s alpha for the scale among MSM in this study was 0.93, which was satisfactory.

#### Self-stigma

The Self-Stigma Scale–Short Form (SSS-S)^42^ was adopted in this study to measure the internalized stigma that participants may have towards themselves as a result of their sexual minority status. Respondents were asked to rate the extent to which they agreed with the statements on a 4-point Likert-type scale, from 1 (strongly disagree) to 4 (strongly agree). The scale consists of 9 items assessing affective, behavioural, and cognitive dimensions of self-stigma. The scale score and each dimension’s scores were based on averaging the item scores, with a higher score indicating a stronger sense of self-stigma. The scores ranged from 1 to 4. The reliability of this instrument in our study was satisfactory, with alpha equal to 0.96.

#### Childhood abuse

We measured two aspects of childhood abuse, physical abuse and psychological abuse. We measured these with 2 questions developed from previous research on childhood abuse^27,29,39^. The two questions assessed whether an individual had ever experienced physical abuse and/or verbal abuse before 17 years of age (yes *vs*. no). Physical abuse was defined as the intentional use of physical force against an individual that results in harm to his/her health, survival, development or dignity. This definition includes hitting, kicking, shaking, biting, burning, poisoning or any other physical behaviour meant to hurt or injure an individual. Psychological abuse was defined as experiencing mistreatment, such as intimidation, threats, humiliation, belittling, ridiculing or verbal abuse, that makes an individual feel serious emotional harm. The Cronbach’s alpha was 0.74 suggesting acceptable internal consistency.

#### HIV-related knowledge

HIV-related knowledge was measured with an 8-item set of questions commonly used for measuring HIV knowledge^43^, which covers HIV transmission routes and modes of prevention. Respondents were given the option to state whether each statement was true or false. The total number of correct answers was computed as the HIV-related knowledge score. A higher score indicated higher levels of knowledge. The reliability of this measure in our study was acceptable, with alpha equal to 0.78.

#### Community engagement

Two items assessed the level of community engagement in the past year. One item was “How often have you engaged in community activities in the past year?” The three possible answers to this item were never, occasionally, and always. The other item was “How many CBOs have you engaged with during the past year?” The possible answers were grouped into three categories: 0, 1–2, and more than 2. The Cronbach’s alpha of this measure was 0.61 suggesting the moderate reliability.

### Statistical analysis

The proportion of missing data at the variable level ranged from 0.29% to 2.63%, and the median was 0.88%. Missing data were handled by using the EM (Expectation Maximization) method. Means, standard deviations (SD), frequencies, and percentages were used to describe the socio-demographic characteristics, sex role, mental health, homosexual stigma, childhood abuse, community engagement, and UAI of MSM in this study. The association between these variables and UAI was evaluated by simple logistic regression and multivariable logistic regression. The enter procedure was used to evaluate the related factors of UAI after adjusting for significant socio-demographic variables in the multivariable logistic regression model. There existed multicollinearity among the three subscales of SSS-S. In order to adjust for this, we only entered the average score of SSS-S as the measure of overall self-stigma into the multivariate regression model to predict UAI. Values of *p* < 0.05 were considered to be statistically significant. SPSS 19.0 software for Windows was used.
